# The Book World of Medicine and Science

**Published:** 1894-12-15

**Authors:** 


### The Book World of Medicine and Science.

Gangrene of the Limbs following Typhoid Fever. By F. Dawtrey Drewitt, M.D., F.R.C.P. (London: H. K. Lewis. 1894.)

In a small brochure of a dozen pages, Dr. Drewitt describes a case in which this uncommon complication of typhoid fever occurred, and gives references to authors who have already described a similar condition. In most of these cases it would appear that the cause was the inpaction of an embolus, a clot washed down from the cavity of the left ventricle, although sometimes perhaps it might arise from arteritis. In the cases mentioned by Trousseau and Patry, the accident occurred late in the disease, and in most it was peeceded by sudden pain in the limb, such as would be caused by the inpaction of an embolus. Emboli continually occur in the spleen and other internal organs, and although they happen not often to block a main artery in a limb, such an event must now and again take place.

Fruit Culture for Profit, for Farmers, Small Holders, Allotment Hodlers, Cottagers, &c. By C. B. Whitehead, B.A. (Society for Promoting Christian Knowledge. 1894.)

At a time when agricultural depression is so widely spread, one greets with satisfaction any suggestion which seems to offer to the British farmer any chance of improving his present unfortunate condition.

Fruit culture has often been strongly advocated, and doubtless in many places it might be introduced and conducted with profit, for it is well known that we annually import large quantities of apples, pears, and the like, which might be grown as well or even better at home.

The little book before us is intended to provide plain directions for anyone wishing to experiment for himself in fruit culture, and it will certainly be helpful to the novice. Not only does it contain excellent hints as to methods of cultivation, but the financial outlay and probable returns are also discussed.

We notice one or two botanical slips in the book, but these do not impair its practical utility, and it deserves to be recommended to those who wish to know how to grow and how to maintain a fruit garden or farm.

Edible and Poisonous Mushrooms: What to Eat and What to Avoid. By M. C. Cooke, M.A., LL.D. With 18 coloured plates, illustrating 48 species. (Society for Promoting Christian Knowledge. 1894.) Price 3s. 6d.

Dr. Cooke is so well-known as a fungologist that we doubt not that the appearance of this little book, with its admirable illustrations, will induce many to put their faith in his guidance, and thereby experience gastronomic delights to which their more cautious (but not necessarily wiser) neighbours will still remain strangers. Many will certainly hear with surprise that many of the "nasty toadstool-looking things" really deserve to rank high amongst table delicacies, but a trial will soon convince them that such is the case. We cannot personally agree with the praise which Dr. Cooke accords to some of the edible species he describes, but that perhaps is due to a lack of proper education, and it is, after all, merely a matter of taste. Those who, after reading Dr. Cooke's little work (and they should be many) would do well to pay especial attention to the warning he gives in his preamble.

